# A Novel Approach to Identify Polytraumatized Patients in Extremis

**DOI:** 10.1155/2018/7320158

**Published:** 2018-04-17

**Authors:** Lukas L. Negrin, Anna Antoni, Stefan Hajdu, Thomas Heinz

**Affiliations:** Department of Trauma Surgery, Medical University of Vienna, Vienna, Austria

## Abstract

**Introduction:**

Due to the fact that early objective identification of polytraumatized patients in extremis is crucial for carrying out immediate life-saving measures, our objectives were to provide and scrutinize a definition that results in a particularly high mortality rate and to identify predictors of mortality in this group.

**Materials and Methods:**

A polytraumatized patient (ISS ≥ 16) was classified “in extremis” if five out of seven parameters (arterial paCO_2 _ > 50 mmHg, hemoglobin < 9.5 g/dl, pH value < 7.2, lactate level > 4 mmol/l, base excess < −6 mmol/l, shock index > 1, and Horowitz index < 300) were met. By applying this definition, polytraumatized patients (age ≥ 18 years), admitted to our level I trauma center within a time period of three years, were retrospectively allocated to the “in extremis” group and to an age-, gender-, and ISS-matched “non-in extremis” group for comparison.

**Results:**

Out of 64 polytraumatized patients (mean ISS, 43.6), who formed the “in extremis” group, 36 patients (56.3%) died, thus revealing a threefold higher mortality rate than in the matched group (18.9%). Within the “in extremis” group, age and ISS were identified as predictors of mortality.

**Conclusion:**

Our definition might serve as a valuable early warning score or at least an impetus for defining polytraumatized patients in extremis in clinical practice.

## 1. Introduction

Polytrauma victims in extremis (very close to death) present the ultimate challenge to trauma surgeons and anesthesiologists. The prompt and accurate identification of these patients, who require the highest resuscitation efforts, is of pivotal importance to increase survival chances by choosing the appropriate life-saving measures, followed by a damage control approach to their injuries [1]. Unfortunately, the term “in extremis,” which is frequently replaced by the term “unstable” in daily clinical practice, does not refer to a precise, commonly accepted classification; it rather denotes an individual assessment of a severely injured patient who manifests abnormal vital signs. Undoubtedly, a straightforward and objective definition as an early warning score, which is based on parameters routinely recorded within the first few minutes after admission, would be of great clinical benefit, assuring uniformity in patient grading and treatment regimen.

Several parameters referring to hemodynamic and cardiorespiratory instability, which indicate an unfavorable prognosis, have already been presented in the literature. In trauma victims blood loss often causes anemia, which is usually detected by low blood hemoglobin concentration. Moderate anemia may be defined by hemoglobin levels between 8 g/ml and 9.5 g/ml and severe anemia by levels lower than or equal to 8 g/ml [2]. In 4,470 critically ill adults (≥16 years) a more than twofold higher mortality rate among individuals with pretransfusion hemoglobin values lower than 9.5 g/ml has been reported (56.5% versus 24.4%) [3]. Uncontrolled hemorrhagic shock still remains a major cause of preventable death after trauma [4, 5]. It is indicated by the shock index, which is defined by the ratio of heart rate to systolic blood pressure [6], serving as a clinical indicator for transfusion requirements and hemostatic resuscitation [7]. If it exceeds 1 an increased mortality rate has been revealed [7, 8]. In general, polytraumatized patients suffering a hemorrhagic shock are at high risk of severe tissue hypoperfusion [9]. When adequate tissue oxygenation cannot be maintained, the serum lactate level starts to rise [10]. As a trauma victim's perfusion worsens, lactic acid rapidly accumulates. The arterial pH value decreases, resulting in a severe lactic acidosis, which is a common phenomenon in shock patients [11]. In general, acidosis is said to occur when the arterial pH value falls below 7.35 [12]. If the pH value is lower than 7.2 it is referred to as severe [13, 14]. The base excess is used as a surrogate marker for lactic acidosis [15], with values lower than −6 mmol/l indicating a poor patient's prognosis [16]. Furthermore, hypoventilation is a frequent finding in polytraumatized patients [17]. It can be caused by inadequate respirator settings in intubated patients, by limited chest excursion secondary to pain from rib fractures, chest wall contusions, and other injuries, resulting in an inadequate elimination of CO_2_ and thus in an increase of the partial pressure of arterial carbon dioxide (paCO_2_) [18], with paCO_2_ values higher than 50 mmHg defining hypercapnia [19] that compromises cerebral perfusion [20]. Due to alveolar hypoventilation a respiratory acidosis occurs as a result of an acid-base balance disturbance [21]. The Horowitz index is defined by the ratio of arterial oxygen partial pressure (paO_2_) and the percentage or concentration of oxygen (FiO_2_) that an individual inhales [22]. An index lower than 300 is included in the definition of an acute respiratory distress symptom (ARDS) [23], a common complication that also increases the mortality rate in polytraumatized patients [24].

Due to the fact that isolated vital signs are unreliable in the assessment of hemodynamic and/or cardiorespiratory instability [7] we presented a novel definition of polytraumatized patients in extremis including several parameters and hypothesized their suitability as an early warning score for accurately identifying polytrauma victims at the point of death. Because mortality naturally is the most important endpoint in patients with severe life-threatening injuries, our definition can only be relevant, if the mortality rate is significantly higher in the group of polytraumatized patients meeting our criteria of “in extremis” than in an age-, gender-, and ISS-matched group of polytrauma victims not conforming to our definition. Therefore, the objectives of this study were to test our definition for suitability and to identify predictors of mortality in this defined “in extremis” group.

## 2. Material and Methods

Our retrospective analysis was approved by the local institutional review board. All patients suffering blunt trauma, who were directly admitted to our level I trauma center within a time period of three years, were included, if they were at least 18 years old and if their Injury Severity Score (ISS) was equal to or higher than 16. From personal experience we combined seven parameters to a concise definition based on threshold values already presented in the literature (Table 1).

Individuals were classified “in extremis,” if five or more criteria were met at hospital admission. According to probability calculations this specification was suggested to classify approximately 20% of the polytraumatized patients, a proportion that was exactly what we were striving for as we aimed to identify polytrauma victims with the least prospects of survival. Demographic data (age, gender, ISS, ventilation period, length of stay at the intensive care unit, overall length of stay, initial blood gas values, shock index, Horowitz index, and survival) of all polytrauma victims, who were classified “in extremis,” were statistically analyzed. For comparison, patients, who did not meet our definition, were matched according to age, gender, and ISS in an equal number. They were combined to the “non-in extremis” group. Statistical analysis was performed using IBM SPSS Statistics Version 23, 64-bit. Parameters were displayed by mean ± standard deviation (SD) and were compared by means of Student's *t*-tests. The Chi-square test was applied to analyze categorical data. Differences in survival were determined by the log-rank test performed on Kaplan-Meier curves. For Receiver Operating Characteristic (ROC) curves the Area under the Curve (AUC) was calculated and presented with a 95% confidence interval (CI). Cut-off values were determined by the maximum sum of sensitivity and specificity [25]. Finally, to ascertain the effect of each selected parameter on the likelihood that polytraumatized patients have to be rated “in extremis” according to our definition simple logistic regression analyses were performed. In general, significant differences were set at a *p* value < 0.05.

## 3. Results

**Figure 1:**
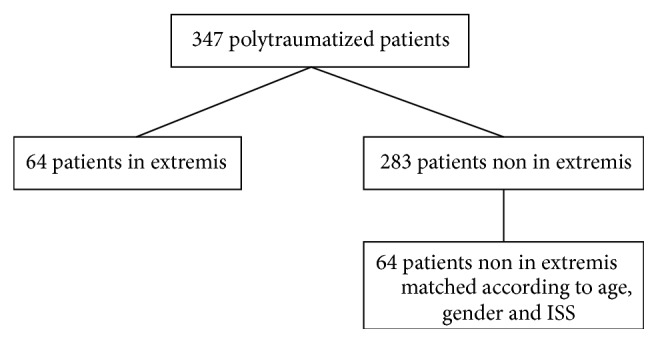
Allocation of polytraumatized patients admitted to our level I trauma center during the observational period.

Out of 347 polytraumatized patients, who met our inclusion criteria, 64 patients (18.4%), 44 men, and 20 women (age, 48.5 ± 21.7 years; ISS, 43.6 ± 21.2) were classified “in extremis” according to our definition (Figure 1).

Their injuries were caused by falls from a great height (22 patients), motor vehicle accidents (18 patients), pedestrian casualties (16 patients), domestic falls (5 patients), and nonmotorized bicycle accidents (3 patients). The most common injured body region was the thorax (75.0%), followed by the lower extremity (67.2%), head (57.8%), upper extremity (50.0%), abdomen (43.8%), spine (35.9%), face (31.3%), and neck (1.6%). The assessment of injury severity according to the Abbreviated Injury Scale (AIS) is presented in Table 2. Solely 18 patients arrived nonintubated. They presented with a mean Glasgow Coma Scale of 8.9 ± 5.3.

**Figure 2:**
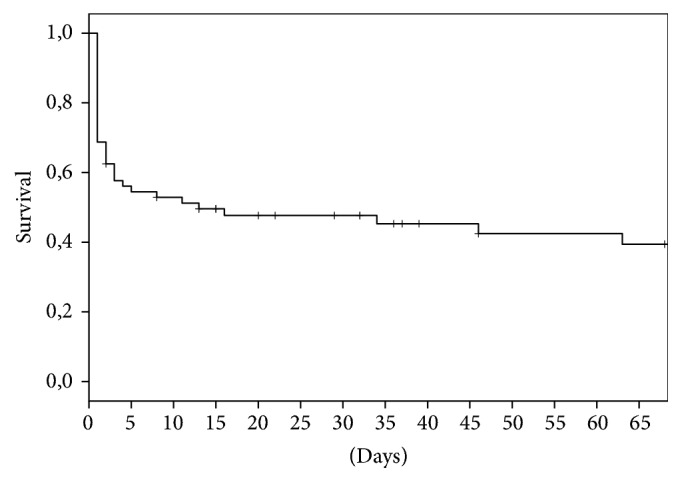
Kaplan-Maier curve referring to the “in extremis” group.

Thirty-six patients (24 men and 12 women) died, including 20 patients (31.3%), who deceased within the first 24 hours, and four patients (6.3%), who deceased on day 2. The mortality rate was 45.3% for the first seven days and 51.6% for the first 30 days. The relevant Kaplan-Meier curve is presented in Figure 2.

Of our study population, 283 polytrauma victims, 210 men and 73 women, did not conform to our definition, thus meeting four of the five criteria at most. Their mean age was 46.3 ± 19.6 years and their mean ISS was 25.9 ± 9.9; 44 patients (15.5%) died. In order to obtain a meaningful comparison with regard to mortality we matched 64 patients classified “non-in extremis,” who presented statistically equivalent distributions according to age, gender and ISS (Table 3).

As anticipated, a significantly higher mortality rate was calculated in polytraumatized patients, who were classified “in extremis” by our definition.

In order to reveal predictors of mortality we subdivided the “in extremis” group into deceased and survivors. Relevant demographic data are presented in Table 4.

**Figure 3:**
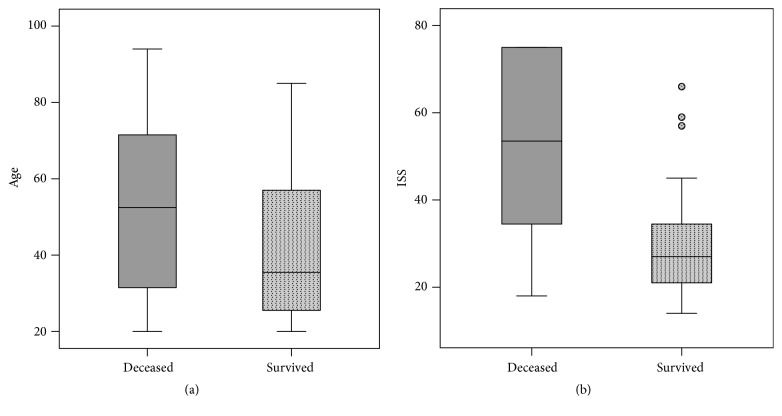
Distribution of age (a) and ISS (b) in the “in extremis” group.

Surprisingly, the comparison of any of the five parameters included in our definition did not show significant differences between the deceased and the survivors in the “in extremis” group. The same applies to the AIS values (*p* > 0.106) and to gender distribution (*p* = 0.683). However, mean age and mean ISS value were significantly higher in the deceased group. The corresponding boxplots are presented in Figure 3.

**Figure 4:**
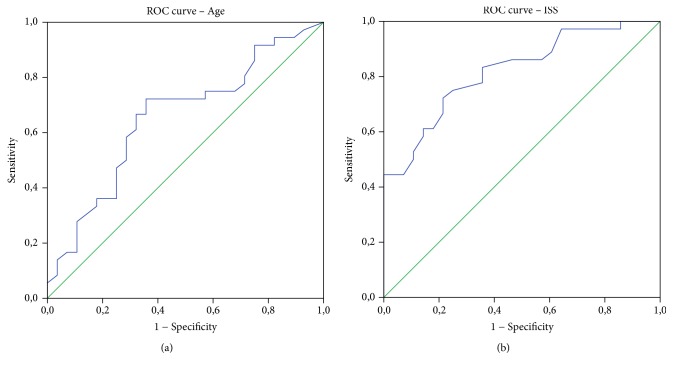
ROC curve for age and mortality (a) and for the ISS value and mortality (b) in the “in extremis” group.

ROC statistics identified age and the ISS value as predictors of mortality in the “in extremis” group, as graphically displayed in Figure 4. For age an AUC of 0.654 (CI, 0.517–0.790; *p* = 0.036) and a cut-off of 40.5 years (sensitivity, 72.2%; specificity, 64.3%) were calculated, whereas an AUC of 0.820 (CI, 0.719–0.929; *p* < 0.0001) and a cut-off of 36 (sensitivity, 72.2%; specificity, 78.6%) were computed for the ISS value.

Of interest, “in extremis” survivors had to stay 16.1 ± 11.1 days at the intensive care unit, where they were ventilated for 7.5 ± 5.2 days. They had to spend 66.1 ± 54.3 days at our trauma center until they were discharged or transferred.

Finally, we aimed to verify the necessity of each of the seven selected parameters for identifying polytrauma victims in extremis immediately after hospital admission. A parameter was coded 1 if its value was within the defined corresponding range (Table 1); otherwise it was coded 0. For each categorized parameter as the sole independent variable a simple logistic regression analysis was performed, whereas the dichotomous rating (1, in extremis; 0, non-in extremis) of our 347 polytraumatized patients served as the dependent variable. By means of the two coefficients of the regression equitation we calculated the likelihood *p* that a polytrauma victim, who meets the inclusion criterion for that particular parameter, will belong to the “in extremis” group. Due to the fact that a predicted probability in the range of 25.9% to 32.0% was calculated for each parameter (Table 5), it can be assumed that each of the seven selected parameters contributes almost equally to our model. Of interest, the test of significance for each of the coefficients in the seven logistic regression models revealed a significance level of <0.001 for each coefficient.

## 4. Discussion

Many years of experience in the field of polytrauma prompted us to present a novel approach to accurately and objectively identify polytrauma victims at the point of death. We hypothesized that a concise definition, based solely on seven items referring to hemodynamic and cardiorespiratory instability, which are available immediately after admission, would act as an early warning score. Retrospectively applying this definition resulted in a patient population, in which every other died.

Our literature search revealed solely four definitions dealing with patients rated “unstable” or “in extremis.” Abrassart et al. [26] defined hemodynamic instability as a combination of hemorrhagic shock, estimated blood loss above 1,500 ml, tachycardia, hypotension (not more than 90 mmHg systolic blood pressure), and delayed capillary refill for at least two seconds. Cardiorespiratory instability was defined by Hravnak et al. [27] and Yousef et al. [28] as a heart rate lower than 40/min or higher than 140/min, a respiration rate lower than 8/min or higher than 36/min, blood pressure lower than 80 or higher than 200 mmHg systolic or higher than 110 mmHg diastolic, and peripheral arterial oxygen saturation less than 85%. The patient status was classified as unstable if any one of the continuously monitored vital signs mentionedabove exceeded the relevant threshold at least once. According to Pape et al. [29] the category “in extremis” includes 12 items referring to the pathophysiological parameters shock (blood pressure < 50–60 mmHg, base excess < −18–(−6) mmol/l, severe acidosis, etc.), coagulation (platelet count < 70,000 *μ*g/ml, etc.), temperature (30°C or less), and soft tissue injuries (Horowitz index < 200, AIS_Thorax_ ≥ 3, etc.). A patient is rated “in extremis” if he/she meets the criteria of at least three pathophysiological parameters. We did not rely on the available definitions for several reasons. Abrassart et al. [26] did not provide an exact definition of hemorrhagic shock, a condition well known in clinical practice but nonuniformly described in the literature [30–33]. Moreover, they did not include parameters referring to respiratory failure. Hravnak et al. [27] and Yousef et al. [28] did not add items describing the acid-base status of blood such as pH value, arterial paCO_2_, or base excess to their definition, although lactic acidosis is commonly caused by hemorrhagic shock and is associated with a poor prognosis [34]. Finally, Pape et al. [29] focused on recommendations for the optimal timing of operative fracture stabilization in blunt trauma victims with orthopedic injuries, aiming to identify those patients, who would suffer great harm by performing definitive fixation of all fractures shortly after admission. Furthermore, coagulation parameters are not available in the first minutes after admission and a body temperature lower than 30°C is rare in polytrauma victims, according to our experience.

Fatalities were the key factor of our retrospective analysis. The overall mortality rate referring to all polytrauma victims admitted to our level I trauma center during the observation period of three years was 23.1%. This percentage is in line with 19.1% [35] and 23.4% [36], recently presented as mortality rates in polytraumatized patients (ISS ≥ 16). Due to the fact that the number of fatalities was threefold higher in the “in extremis” group than in the age-, gender-, and ISS-matched “non-in extremis” group (56.3% versus 18.9%) our novel definition was capable of identifying those individuals, who presented with the worst survival rate. Surprisingly, a multicenter study, conducted at seven US level I trauma centers during a time period of almost six years, reported an incredibly low mortality rate of 16.7% in 1,637 individuals (mean age: 42.4 years; mean ISS: 32.1), who were critically injured by blunt mechanism and suffered an hemorrhagic shock, defined as base excess ≤ −6 mmol/l or systolic blood pressure < 90 mmHg within 60 minutes at hospital admission, requiring blood transfusion within six hours after trauma [32]. Unlike ours, Cuschieri et al. [32] excluded trauma victims with an anticipated survival of less than 24 hours from time to injury. In our “in extremis” group 31.3% deceased within the first 24 hours. Nevertheless, subtracting this percentage from the overall mortality rate of 56.3% results in a rate of 25%, which is still 50% higher than the 16.7% reported by Cuschieri et al. [32]. Although lower patient age and ISS may have a positive impact on survival rate, both parameters can hardly explain this discrepancy, which indicates that our definition is actually restricted to patients at the greatest risk of death and thus can serve as early warning score in clinical practice.

By revealing age and ISS as predictors of mortality in polytraumatized patients in extremis we achieved our second objective. With regard to patient age, our result corresponds to already published findings. According to Vanzant et al. [31] advanced age (≥ 55 years) has to be considered as one of the strongest noninjury related risk factors for poor outcomes in individuals (age ≥ 16 years) suffering severe blunt trauma (ISS ≥ 16) without severe traumatic brain injury, presenting with hemorrhagic shock (systolic blood pressure < 90 mmHg or base excess ≤ −6 mmol/l, and requiring blood transfusion). Kuhne et al. [30] reported a higher mortality rate, independent of injury severity, in polytraumatized patients (ISS ≥ 16) with a minimum age of 56 years compared to the 15 to 55 age group. Finally, with regard to injury severity, our cut-off value of 36 for the ISS as a predictor of mortality is in line with the results of Strnad et al. [9], who presented an ISS value of 30 for severely injured intubated blunt trauma patients.

Limitations of our study include its single center and retrospective design. Our results are based on the analysis of prerecorded data of one level I trauma center that were originally assessed and collected for reasons other than research. Furthermore, selection bias cannot be ruled out for the matched control group.

## 5. Conclusions

Our allocation of polytraumatized patients to an “in extremis” group and an age-, gender-, and ISS-matched “non-in extremis” group according to our definition, which is based on seven parameters dealing with hemodynamic and cardiorespiratory instability, has resulted in a threefold higher mortality rate in the “in extremis” group. Polytrauma victims in extremis, more than 40 years of age, presenting with an ISS higher than or equal to 36 have the worst prognosis of survival. Our novel approach to identify polytrauma victims at the highest risk of death immediately after admission might serve as a helpful tool in clinical practice. At least, it might be considered as an impetus for further prospective investigations as part of multicenter studies.

## Figures and Tables

**Table 1:** Definition of polytraumatized patients in extremis.

Arterial paCO_2_	>50 mmHg
Hemoglobin	<9.5 g/dl
pH value	<7.2
Lactate level	>4 mmol/l
Base excess	<−6 mmol/l
Shock index	>1
Horowitz index	<300

**Table 2:** Abbreviated Injury Scale in the “in extremis” group.

	Mean ± SD

Thorax	2.66 ± 1.85
Head	2.27 ± 2.19
Lower extremities	2.08 ± 1.78
Abdomen	1.50 ± 1.89
Spine	1.09 ± 1.73
Upper extremities	0.97 ± 1.10
Face	0.59 ± 0.99
Neck	0.02 ± 0.13

**Table 3:** Comparison of the “in extremis” group and the matched “non-in extremis” group.

	In extremis	Non-in extremis	*p* value
Age (mean ± SD)	48.5 ± 21.7	45.1 ± 21.1	0.377
Gender (male : female)	44 : 20	46 : 18	0.597
ISS (mean ± SD)	43.6 ± 10.9	39.8 ± 21.24	0.201
Mortality rate	56.3%	18.9%	<0.001

**Table 4:** Demographic data (mean ± SD) referring to the “in extremis” group.

	Total	Deceased	Survivors	*p* value
Age (years)	48.5 ± 21.7	53.4 ± 21.9	42.1 ± 20.1	0.037
ISS	43.6 ± 21.2	53.7 ± 20.9	30.6 ± 13.3	<0.001
Arterial paCO_2_ (mmHg)	55.5 ± 10.8	56.4 ± 10.8	54.2 ± 10.9	0.549
Hemoglobin (g/dl)	9.26 ± 1.8	8.8 ± 1.4	9.7 ± 2.2	0.129
pH value	7.14 ± 0.16	7.10 ± 0.17	7.16 ± 0.14	0.476
Lactate level (mmol/l)	6.66 ± 4.58	7.34 ± 4.34	5.76 ± 4.86	0.305
Base excess (mmol/l)	−10.4 ± 5.8	−10.6 ± 6.3	−10.0 ± 5.1	0.742
Shock index	1.4 ± 0.4	1.5 ± 0.4	1.4 ± 0.4	0.671
Horowitz index	228.78 ± 96.84	228.6 ± 86.8	229.0 ± 105.8	0.971

**Table 5:** Coefficients in the logistic regression equitation.

logit *p* = *a* · *x* + *b*			
Parameter *x*	*a*	*b*	*p* (*x* = 1)
paCO_2_	1.791	−2.842	0.259
Hemoglobin	1.726	−2.514	0.313
pH value	2.331	−3.117	0.313
Lactate level	1.538	−2.573	0.262
Base excess	2.065	−2.821	0.259
Shock index	1.621	−2.376	0.320
Horowitz index	1.080	−2.001	0.285
